# NADPH‐dependent 5‐keto‐D‐gluconate reductase is a part of the fungal pathway for D‐glucuronate catabolism

**DOI:** 10.1002/1873-3468.12946

**Published:** 2017-12-30

**Authors:** Joosu Kuivanen, Peter Richard

**Affiliations:** ^1^ VTT Technical Research Centre of Finland Ltd Espoo Finland

**Keywords:** 5‐keto‐gluconate, *Aspergillus niger*, CRISPR/Cas9, D‐gluconate‐5‐dehydrogenase, D‐glucuronate, EC 1.1.1.69, fungal pathway

## Abstract

NADPH‐dependent 5‐keto‐D‐gluconate reductase was identified as a missing element in the pathway for D‐glucuronate catabolism in fungi. The disruption of the gene, *gluF*, by CRISPR/Cas9 in the filamentous fungus *Aspergillus niger* resulted in a strain unable to catabolise D‐glucuronate. The purified GluF protein was characterized and *k*_cat_ and *K*_m_ values of 23.7 ± 1.8 s^−1^ and 3.2 ± 0.1 mm for 5‐keto‐D‐gluconate, respectively, were determined. The enzyme is reversible and is active with NADP^+^ and D‐gluconate. We suggest a pathway for D‐glucuronate catabolism with the intermediates L‐gulonate, 2‐keto‐L‐gulonate, L‐idonate, 5‐keto‐D‐gluconate, D‐gluconate and D‐gluconate‐6‐phosphate which is a part of the pentose phosphate pathway. A fungal enzyme activity for the conversion of L‐gulonate to 2‐keto‐L‐gulonate remains to be identified.

## Abbreviations

**D‐glcUA**, D‐glucuronate

**gDNA**, genomic DNA

**NHEJ**, nonhomologous end joining

The filamentous fungus *Aspergillus niger* is a versatile decomposer of biomass polymers and an important source of biomass‐hydrolysing enzymes in industrial biotechnology 1. After hydrolysis, a wide range of different sugars and sugar acids are released from plant biomass and catabolised by the fungus. The most common metabolic pathways which catabolise biomass constituents are known; however, catabolic pathways of some less abundant sugars and sugar acids remain still unknown not only in *A. niger* but also in other saprotrophic microorganisms. Discovery and characterization of these unknown metabolic pathways may serve as a source of biochemical reactions for biotechnological applications.

**Figure 1:**
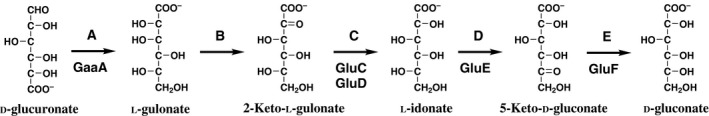
Hypothetical fungal pathway for D‐glucuronate catabolism. (A) NADPH:D‐glucuronate reductase GaaA, that is a bifunctional enzyme that also catalyses the reduction in D‐galacturonic acid. (B) Hypothetical reaction: an enzyme for L‐gulonate 2‐dehydrogenase has not been identified. (C) 2‐keto‐L‐gulonate is reduced by two enzymes, the NADH‐requiring GluC and the NADPH‐requiring GluD. (D) The L‐idonate 5‐dehydrogenase, GluE, uses NAD^+^ as a cofactor. (E) The NADPH‐requiring 5‐keto‐D‐gluconate reductase GluF described in this study.

D‐Glucuronate (D‐glcUA) is a biomass component occurring, for example, in the plant cell wall polysaccharide glucuronoxylan 2 and in the algal polysaccharide ulvan 3. Until recently, only two bacterial catabolic pathways – an oxidative 4, 5 and an isomerase pathway 6 – and an animal pathway 7 were known to catabolise D‐glcUA. In the bacterial oxidative pathway, D‐glcUA is oxidised to D‐glucarate and with the aid of dehydratase and aldolase activities converted to pyruvate and D‐glyceraldehyde. In the bacterial isomerase pathway, the metabolites are D‐fructuronate, D‐mannonate, keto‐deoxy‐gluconate and keto‐deoxy‐gluconate‐6‐phosphate and the products are pyruvate and glyceraldehyde‐3‐phosphate. In the animal pathway, D‐glcUA is reduced to L‐gulonate which is then oxidised to 3‐keto‐L‐gulonate. A decarboxylase converts the 3‐keto‐L‐gulonate to L‐xylulose which is then further converted to D‐xylulose‐5‐phosphate which is a part of the pentose phosphate pathway. Recently, the first steps of the fungal catabolic D‐glcUA pathway were described (Fig. 1). The pathway, which is distinctly different to all the other pathways, begins with the reduction in D‐glcUA to L‐gulonate that is further oxidized to 2‐keto‐L‐gulonate in the second reaction 8. The first reaction is catalysed not only by the uronic acid reductase GaaA, which is also a part of the fungal D‐galacturonic acid pathway, but also by another still unknown reductase 8, 9. The gene encoding the enzyme for the second step – oxidation of L‐gulonate to 2‐keto‐L‐gulonate – is still unknown. In the third step, 2‐keto‐L‐gulonate is reduced to L‐idonate by two different enzymes, the GluC using NADH and by GluD using NADPH as a cofactor 8, 10. Deletion of *gluC* caused deficiency in D‐glcUA catabolism completely while *gluD* deletion resulted in accumulation of 2‐keto‐L‐gulonate. The fourth step in the fungal pathway is an oxidation of L‐idonate to 5‐keto‐D‐gluconate by the action of NAD^+^‐dependent oxidoreductase GluE 10. Deletion of *gluE* gene resulted in a deficient phenotype in D‐glcUA catabolism in *A. niger*. The subsequent steps in the fungal D‐glcUA pathway from 5‐keto‐D‐gluconate are not known.

In the present study, we describe the fifth step of the fungal D‐glcUA pathway. The 5‐keto‐D‐gluconate formed by the action of GluE is subsequently reduced to D‐gluconate by the action of oxidoreductase we called GluF.

## Materials and methods

### Strains

The *Aspergillus niger* strain ATCC 1015 (CBS 113.46) was used as a wild‐type (*wt*) and parental strain for *gluF* deletion. All the plasmids were constructed in *Escherichia coli* TOP10 cells. A modified *Saccharomyces cerevisiae* strain CEN.PK2 (*MATa, leu2‐3/112, ura3‐52, trp1‐289, his3‐11, MAL2‐8c, SUC2*) was used for the production and purification of GluF enzyme.

### Media and cultural conditions

Luria Broth culture medium supplemented with 100 μg·mL^−1^ ampicillin and cultural conditions of 37 °C and 250 r.p.m. were used with *E. coli*. YPD medium (10 g·L^−1^ yeast extract, 20 g·L^−1^ peptone and 20 g·L^−1^ D‐glucose) was used for yeast precultures. After the transformation of an expression plasmid in yeast, SCD‐URA (uracil deficient synthetic complete media supplemented with 20 g·L^−1^ D‐glucose) plates were used for uracil auxotrophic selection. SCD‐URA medium was used in protein production. All the yeast cultivations were carried out at 30 °C and the liquid cultivations at 250 r.p.m. *A. niger* spores were generated on potato dextrose plates and ~ 10^8^ spores were inoculated to 50 mL of YP medium (10 g·L^−1^ yeast extract, 20 g·L^−1^ peptone) containing 30 g·L^−1^ gelatin for precultures. Mycelia were pregrown in 250‐mL Erlenmeyer flasks by incubating overnight at 28 °C, 200 r.p.m. and harvested by vacuum filtration, rinsed with sterile water and weighted. In the deletion of *gluF* from *A. niger* wt and in the complementation of the resulting strain, *A. nidulans* defined minimal medium 11 supplemented with 1.2 m D‐sorbitol, 400 μg·mL^−1^ hygromycin and 20 g·L^−1^ agar (pH 6.5) were used. The defined minimal medium was used in the phenotypic characterization in liquid cultivations and contained 20 g·L^−1^ D‐glcUA. The pH was adjusted to 6.5. These cultures were inoculated with 8 g·L^−1^ (dry mass) of mycelia which was pregrown on YP medium containing 20 g·L^−1^ D‐xylose and were cultivated in 24‐well plates in 4 mL final volume. Agar plates used for phenotypic characterization contained SC medium (synthetic complete), 15 g·L^−1^ agar and 20 g·L^−1^ D‐glcUA or D‐glucose. Plates were inoculated with 3 × 10^6^ spores.

### Transcriptional analysis

The RNA sequencing for transcriptional analysis was carried out previously 8. In brief, *A. niger wt* strain was precultivated on YP‐gelatin medium and transferred to defined minimal medium containing D‐glcUA as sole carbon source. Samples were collected after 0 and 4 h by vacuum filtration and frozen with liquid nitrogen. RNeasy Plant Mini Kit (Qiagen) was used for total RNA extraction. RNA sequencing was carried out by GATC (Constance, Germany) and the sequencing data were processed as described earlier 8.

### Protein production and purification

The *gluF* gene was custom synthesized as a yeast codon‐optimized gene (GenScript, USA), digested with *EcoRI* and *BamHI* (both NEB) and ligated into the modified pYX212 plasmid 12 containing *TPI1* promoter and *URA3* selectable marker. For the histidine‐tagged protein, *gluF* was amplified by PCR with the primers P1 and P2 (Table 1) and ligated in a similar manner to the modified pYX212 plasmid. A yeast strain was then transformed with the resulting plasmids using the lithium acetate method 13. The procedure for protein production and purification was described previously 8.

**Table 1:** Oligonucleotides and sgRNA used in the study

Name	Sequence	Description
P1	TATAGAATTCACCATGcatcaccatcaccatcacGGTGGCGGTATGTCTGCCGCTGTCGCAT	gluF ORF amplification with His‐tag, EcoRI site, fwd primer
P2	TATAGGATCCTTATCTACCCATCC	gluF ORF amplification with His‐tag, BamHI site, rev primer
P3	GAGCTATCAGTGAGAACGAT	gluF 5′, 609 bp up from the strart codon, fwd primer
P4	ACTCTGACTCATCTCGCGCC	gluF 3′, 600 bp down from gRNA PAM sequence, rev primer
P5	TATATAGAGCTCCACCATGTCGGCAGCAGTTGCGTC	Amplification of gluF ORF, SacI flank, fwd primer
P6	TATATACCCGGGTCACCGTCCCATCCAACCGC	Amplification of gluF ORF, XmaI flank, rev primer
gluF sgRNA	GCGTCGCTATTTTCCCTCAG	gluF sgRNA protospacer sequence

### *In vitro* reaction and enzymatic assays

The oxidoreductase activity of purified GluF protein was assayed using Konelab 20XT Clinical Chemistry Analyzer (Thermo Electron Oy, Finland). The reaction mixture contained 50 mm Tris buffer, 400 μm NADP^+^ or NADPH, a substrate in different concentrations and purified proteins in a final concentration of 3.6 mg·L^−1^. The sugar acid library that was used contained D‐galacturonate, D‐glucuronate, L‐galactonate, L‐gulonate, 2‐keto‐L‐gulonate, L‐idonate, 5‐keto‐D‐gluconate, meso‐galactarate, D‐tagaturonate and D‐gluconate. The pH 8 was used with NADP^+^ and D‐gluconate and pH 7 with NADPH and 5‐keto‐D‐gluconate. The reaction was started by addition of the purified protein and the formation/consumption of NADPH was followed at 340 nm. The kinetic parameters were determined using the IC50 tool kit (www.ic50.tk). The *in vitro* reaction experiment for HPLC analysis contained 50 mm Tris buffer pH 7, 10 mm NADPH, 10 mm 5‐keto‐D‐gluconate and 3.6 mg·L^−1^ purified GluF.

### Gene deletions and insertions in *A. niger*

The *gluF* gene was disrupted from *A. niger wt* strain using CRISPR/Cas9‐mediated double‐stranded cleavage to the genomic DNA at the *gluF* open reading frame and the following native non‐homologous end joining (NHEJ) repair mechanism. The plasmid expressing Cas9 and the sgRNA was based on the pFC‐332 14. The DNA cassette containing *gpdA* promoter (*gpdAp*), sgRNA for *gluF* (Table 1) with ribozyme elements and *trpC* terminator was designed as described by Nodvig *et al*. 14. The *gluF* sgRNA cassette was ordered as gBlock (IDT) and was ligated to *PacI* site of pFC‐322. The resulting plasmid was transformed to *A. niger wt* strain and was selected for growth in the presence of hygromycin. Few resulting colonies from the transformation were screened for the *gluF* gene disruption with colony PCR using Phire direct PCR kit (Thermo Scientific) and the primers P3 and P4 (Table 1). The gDNA of the selected clone was sequenced at *gluF* region with the primers P3 and P4 and the exact *gluF* gene disruption was determined. The resulting strain *∆gluF* was restreaked several times onto YPD plates without hygromycin in order to remove the Cas9‐ and sgRNA‐expressing plasmid. The resulting colonies were screened for inability to grow in the presence of hygromycin. The deletion of *gluF* was complemented by integrating an expression cassette containing the native *gluF*‐encoding sequence under the *gpdAp*. The expression cassette was constructed by amplifying *gluF* from gDNA with the primers P5 and P6 (Table 1). The resulting fragment was digested with *SacI* and *XmaI* and was ligated into a *SacI*‐ and *XmaI*‐digested pRS426‐derived plasmid JKp‐*hph* 15 between *gpdAp* and *trpC* terminator. The resulting plasmid contained selection marker for hygromycin. The plasmid was linearized with *DraI* and was transformed into *∆gluF* strain. The cassette was randomly integrated into the genome by selecting growth on hygromycin plates.

### Chemical analyses

Samples were removed from liquid cultivations at intervals and mycelium was separated from the supernatant by filtration. The concentrations of D‐glcUA, 5‐keto‐D‐gluconate and D‐gluconate in supernatants and *in vitro* reaction mixture were determined by HPLC using a Fast Acid Analysis Column (100 mm × 7.8 mm, BioRad Laboratories, Hercules, CA) linked to an Aminex HPX‐87H organic acid analysis column (300 mm × 7.8 mm, BioRad Laboratories) with 5.0 mm H_2_SO_4_ as eluent and a flow rate of 0.5 mL·min^−1^. The column was maintained at 55 °C. Peaks were detected using a Waters 2487 dual wavelength UV (210 nm) detector.

## Results

### The transcription of gluF gene is induced on D‐glcUA and the deletion of the gene has an effect on D‐glcUA catabolism

**Figure 2:**
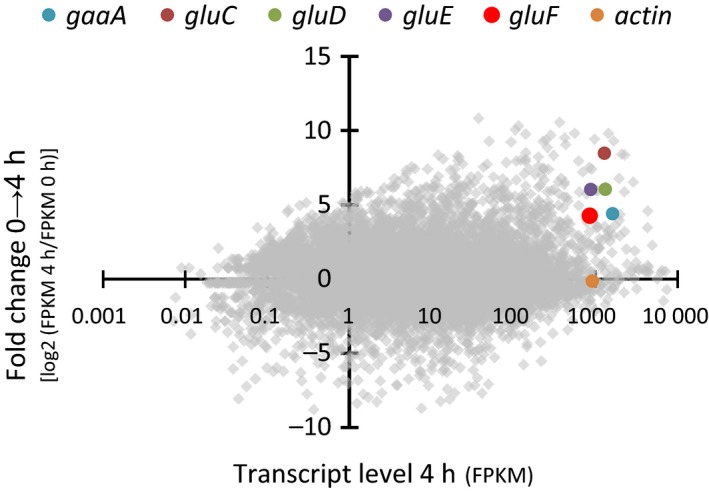
RNA sequencing of *A. niger* ATCC1050 wild‐type strain after transferred to D‐glucuronate medium. Data shown as fold change in transcript levels between 0 and 4 h samples (*y*‐axis) and as absolute transcript levels (*x*‐axis) 4 h after the transfer to D‐glucuronate. Putative genes with significantly increased transcript levels after the transfer to D‐glcUA (> 1) and absolute transcript levels similar or higher than the value of actin were examined in more detail. The genes *gaaA*,* gluC*,* gluD*,* gluE*,* gluF* and actin are highlighted. Transcript levels are presented as fragments per kilobase of exon per million fragments mapped (FPKM).

The transcriptome of *A. niger wt* strain cultivated on D‐glcUA was sequenced in the previous studies 8, 10. Putative genes were evaluated based on altered transcript levels after the shift to D‐glcUA and prediction of protein function. Among the characterized genes involved in the catabolism of D‐glcUA – *gaaA*,* gluC*,* gluD* and *gluE* – a gene with the JGI protein ID 1156802 (JGI, MycoCosm, *A. niger* ATCC 1015 v.4.0 database) (GenBank: EHA27001.1) that we named that *gluF* has an induced transcript level (Fig. 2). The predicted protein product of *gluF* is 265 amino acids long and belongs to the family of short‐chain dehydrogenases/reductases.

In order to investigate the possible function of *gluF* for D‐glcUA catabolism, the gene was disrupted from *A. niger wt* strain. We used the plasmid‐based CRISPR/Cas9 approach described by Nodvig *et al*. 14 in which Cas9 and sgRNA are both expressed from the same AMA plasmid and no donor DNA is used to fix the resulting double‐stranded brake in the genomic target. The resulting gene disruption is based on the NHEJ pathway‐mediated repair which typically introduces short insertions or deletions to the CRISPR target site. After the transformation, a few colonies were screened using diagnostic PCR. One of the colonies revealed to have a deletion of 489 bp in the *gluF* gene disrupting the start codon (Fig. S1). We selected this *∆gluF* clone for further studies. In addition, a complemented strain based on *∆gluF* but having *gluF* expressed under *gpdAp* (*∆gluF‐gpdAp‐gluF*) was generated.

**Figure 3:**
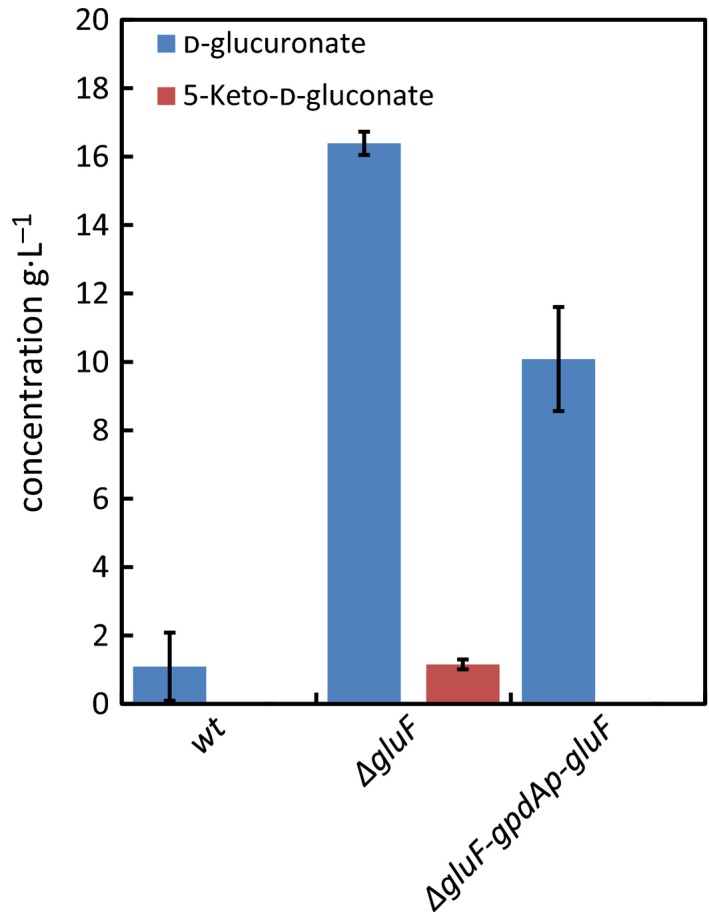
D‐Glucuronate and 5‐keto‐D‐gluconate concentrations in liquid cultivations after 64 h with *A. niger wt*,* ∆gluF* and *∆gluF*‐*gpdAp‐gluF*. Data represent means ± standard deviation from three biological repeats.

**Figure 4:**
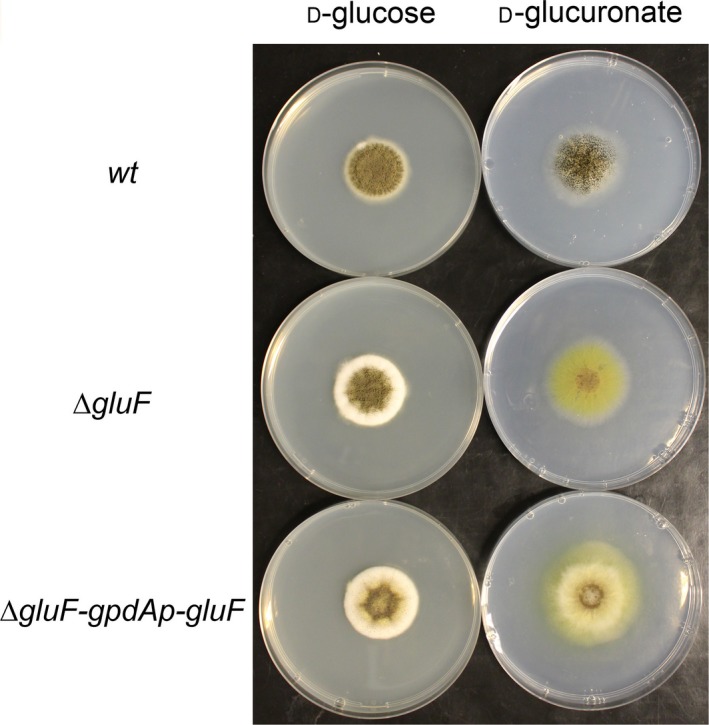
*A. niger wt*,* ∆gluF* and *∆gluF‐gpdAp‐gluF* strains cultivated on plates with D‐glucose or D‐glucuronate as sole carbon source.

The ability of *∆gluF* strain to catabolise and grow on D‐glcUA was investigated in liquid cultivations (Fig. 3) and on plates (Fig. 4). Precultivated mycelia of *wt* strain, *∆gluF* and *∆gluF‐gpdAp‐gluF* were transferred to medium containing 20 g·L^−1^ D‐glcUA and the concentration was determined after 64 h (Fig. 3). As shown earlier 8, 10, the *wt* strain is able to catabolise D‐glcUA, however, in *∆gluF* strain significantly disturbed ability to catabolise D‐glcUA was observed. In addition, accumulation of 5‐keto‐D‐gluconate, a putative intermediate metabolite in the fungal D‐glcUA pathway, was observed in the cultivation medium by *∆gluF* strain. The complemented strain *∆gluF‐gpdAp‐gluF* did not accumulate 5‐keto‐D‐gluconate and had utilized about 50% of the initial D‐glcUA after 64 h. Also, the growth of *∆gluF* on D‐glcUA plates was disturbed compared to *wt* and the strain was not able to sporulate on the plate (Fig. 4). The ability to grow on D‐glcUA plates was partially restored in the complemented strain *∆gluF‐gpdAp‐gluF*.

### The gene gluF codes for NADPH‐dependent 5‐keto‐D‐gluconate reductase

**Figure 5:**
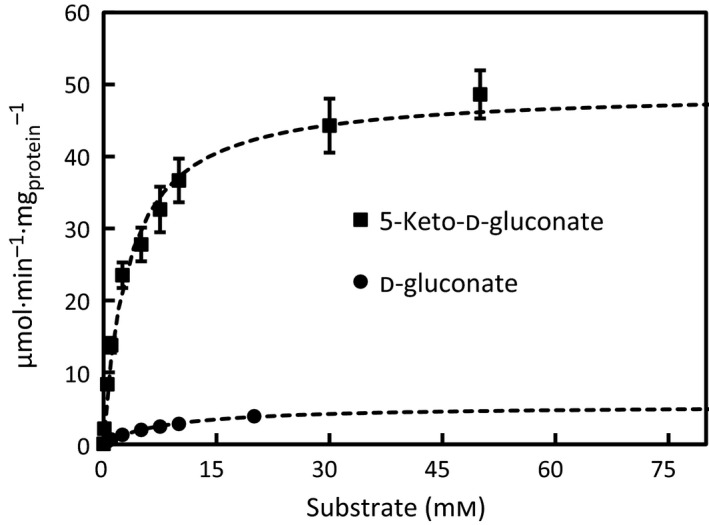
Oxidoreductase activity of the purified GluF protein with NADPH towards 5‐keto‐D‐gluconate and with NADP^+^ towards D‐gluconate. Data represent means ± standard deviation from three technical repeats.

GluF protein was produced in yeast and purified for detailed characterization. In the preliminary tests, the oxidoreductase activity of GluF was screened from the yeast‐expressing *gluF* using the crude extract and a small library of sugars and sugar acids (data not shown). The preliminary screen revealed a strictly NADPH‐dependent activity of GluF towards 5‐keto‐D‐gluconate and NADP^+^‐dependent activity towards D‐gluconate. The activity towards 5‐keto‐D‐gluconate was also tested with NADH; however, this resulted in no detectable oxidoreductase activity. In the next step, the histidine‐tagged GluF protein was produced and purified for further characterization. In order to confirm that the reaction product from 5‐keto‐D‐gluconate is D‐gluconate, an *in vitro* reaction of GluF with 5‐keto‐D‐gluconate and NADPH was analysed using HPLC (Fig. S2). The analysis confirmed that GluF indeed catalysed an NADPH‐dependent reaction between 5‐keto‐D‐gluconate and D‐gluconate. The kinetic parameters of GluF were determined in more detail with the purified enzyme (Fig. 5) and are summarized in Table 2. As a result, affinity towards 5‐keto‐D‐gluconate and maximum reaction rate from 5‐keto‐D‐gluconate to D‐gluconate was at significantly higher level than the values of the reverse reaction from D‐gluconate to 5‐keto‐D‐gluconate.

**Table 2:** Kinetic parameters of the purified GluF protein. Data represent means ± standard deviation from three technical repeats

Protein	Substrate	*V*_max_ (μmol·min^−1^ mg^−1^)	*K*_m_ (mm)	*k*_cat_ (s^−1^)
GluF	5‐keto‐D‐gluconate	49.1 ± 3.8	3.2 ± 0.1	23.7 ± 1.8
	D‐gluconate	5.4 ± 0.5	8.4 ± 0.1	2.6 ± 0.2

## Discussion

We identified the first fungal gene coding for a NADPH‐requiring 5‐keto‐D‐gluconate reductase. The gene with the GenBank accession number EHA27001.1 was wrongly annotated as a 2‐deoxy‐D‐gluconate 3‐dehydrogenase. It is the first description of such a gene in a eukaryotic organism; however, the enzyme activity was described previously. In the fungus *Penicillium notatum,* an NADPH‐requiring 5‐keto‐D‐gluconate reductase activity was described after growth on 5‐keto‐D‐gluconate 16.

Our results suggest that the NADPH‐requiring 5‐keto‐D‐gluconate reductase is a part of the pathway for D‐glcUA catabolism. The deletion of the corresponding gene *gluF* stops D‐glcUA catabolism and the purified enzyme catalyses the reversible reaction from 5‐keto‐D‐gluconate and NADPH to D‐gluconate and NADP^+^. With the 5‐keto‐D‐gluconate reductase, the fungal pathway for D‐glcUA catabolism is almost complete, only the step from L‐gulonate to 2‐keto‐gulonate is elusive. An L‐gulonate 2‐dehydrogenase has not been described in the literature, and it might be that reaction is more complex with more than one enzyme involved.

A 5‐keto‐D‐gluconate reductase, EC 1.1.1.69, has been described as a bacterial enzyme that can use NADH or NADPH as a cofactor (www.brenda-enzymes.org). Also, *E. coli* activities with NADH and NADPH were described 17. A gene for the 5‐keto‐D‐gluconate reductase, *idnO*, that is NADPH specific, was described in *E. coli* as being part of a pathway for L‐idonate catabolism 18. In the pathway, an L‐idonate 5‐dehydrogenase is oxidising the L‐idonate to 5‐keto‐D‐gluconate and the 5‐keto‐D‐gluconate is subsequently reduced to D‐gluconate. D‐Gluconate is then phosphorylated, and the resulting 6‐phosphogluconate enters the Entner–Doudoroff pathway 18. The steps from L‐idonate to D‐gluconate are similar in *E. coli* and in *A. niger;* however, the bacterial pathway for D‐glcUA catabolism is distinctly different to the fungal pathway and does not have L‐idonate or D‐gluconate as an intermediate metabolite. This is the case for the isomerase pathway not only for D‐glcUA catabolism, for example, *E. coli* but also for the oxidative pathway. Both 5‐keto‐D‐gluconate reductases, from *E. coli* and *A. niger*, are the members of the short‐chain dehydrogenase protein family; however, the similarity in protein sequence is below 35%.

The GluF connects the D‐glcUA pathway with the pathway of D‐gluconate catabolism. Under some conditions, *A. niger* oxidizes extracellular D‐glucose to D‐gluconate which is then taken up and catabolised further through the phosphorylation to D‐gluconate‐6‐phosphate and subsequently via pentose phosphate pathway. In fungal microorganisms, D‐gluconate is generally believed to be catabolised through the pentose phosphate pathway and not through the Entner–Doudoroff pathway as in *E. coli*. A kinase for the phosphorylation of D‐gluconate to D‐gluconate‐6‐phosphate, that is a metabolite of the pentose phosphate pathway, was described in *A. niger* 19. It was however also suggested that some strains of *A. niger* catabolise D‐gluconate through the nonphosphorylative Entner–Doudoroff pathway including dehydratation of D‐gluconate to 2‐keto‐3‐deoxy‐gluconate which is then split to D‐glyceraldehyde and pyruvate by the action of an aldolase 20, 21.

To conclude, this study is the first description of a eukaryotic gene‐encoding 5‐keto‐D‐gluconate reductase. In addition, the study indicates that the gene has a function in the fungal catabolic D‐glcUA pathway which would connect D‐glcUA catabolism to the pentose phosphate pathway.

## Author contributions

JK and PR designed and JK performed the experiments. JK and PR wrote the manuscript.

## Supporting information


**Fig. S1.** Disruption of the *gluF* gene after CRISPR/Cas9 genome editing.

**Fig. S2.** HPLC analysis of the reaction mixtures containing TRIS‐buffer, NADPH and 5‐keto‐D‐gluconate (A) without, (B) with the purified GluF protein and (C) D‐gluconate standard solution.

## References

[1] de Vries RP and Visser J (2001) Aspergillus enzymes involved in degradation of plant cell wall polysaccharides. Microbiol Mol Biol Rev 65, 497–522. 11729262 10.1128/MMBR.65.4.497-522.2001 PMC99039

[2] Reis D , Vian B and Roland J‐C (1994) Cellulose‐glucuronoxylans and plant cell wall structure. Micron 25, 171–187.

[3] Lahaye M and Robic A (2007) Structure and function properties of Ulvan, a polysaccharide from green seaweeds. Biomacromol 8, 1765–1774. 10.1021/bm061185q 17458931

[4] Dagley S and Trudgill PW (1965) The metabolism of galactarate, D‐glucarate and various pentoses by species of Pseudomonas. Biochem J 95, 48–58. 14333567 10.1042/bj0950048 PMC1215176

[5] Chang YF and Feingold DS (1970) D‐Glucaric acid and galactaric acid catabolism by Agrobacterium tumefaciens. J Bacteriol 102, 85–96. 4314480 10.1128/jb.102.1.85-96.1970 PMC284973

[6] Ashwell G (1962) Enzymes of glucuronic and galacturonic acid metabolism in bacteria. Methods Enzymol 5, 190–208.

[7] Hankes L , Politzer W , Touster O and Anderson L (1969) Myo‐inositol catabolism in human pentosurics: the predominant role of the glucuronate‐xylulose‐pentose phosphate pathway. Ann N Y Acad Sci 165, 564–576. 5259614

[8] Kuivanen J , Sugai‐Guérios MH , Arvas M and Richard P (2016) A novel pathway for fungal D‐glucuronate catabolism contains an L‐idonate forming 2‐keto‐L‐gulonate reductase. Sci Rep 6, 26329. 27189775 10.1038/srep26329 PMC4870679

[9] Martens‐Uzunova ES and Schaap PJ (2008) An evolutionary conserved D‐galacturonic acid metabolic pathway operates across filamentous fungi capable of pectin degradation. Fungal Genet Biol 45, 1449–1457. 18768163 10.1016/j.fgb.2008.08.002

[10] Kuivanen J , Arvas M and Richard P (2017) Clustered genes encoding 2‐keto‐l‐gulonate reductase and l‐idonate 5‐dehydrogenase in the novel fungal d‐glucuronic acid pathway. Front Microbiol 8, 1–10. 28261181 10.3389/fmicb.2017.00225 PMC5306355

[11] Barratt R , Johnson G and Ogata W (1965) Wild‐type and mutant stocks of Aspergillus nidulans. Genetics 52, 233–246. 5857598 10.1093/genetics/52.1.233 PMC1210840

[12] Verho R , Putkonen M , Londesborough J , Penttilä M and Richard P (2004) A Novel NADH‐linked L‐xylulose reductase in the L‐arabinose catabolic pathway of yeast. J Biol Chem 279, 14746–14751. 14736891 10.1074/jbc.M312533200

[13] Gietz R and Schiestl R (2007) High‐efficiency yeast transformation using the LiAc/SS carrier DNA/PEG methode. Nat Protoc 2, 31–34. 17401334 10.1038/nprot.2007.13

[14] Nodvig CS , Nielsen JB , Kogle ME and Mortensen UH (2015) A CRISPR‐Cas9 system for genetic engineering of filamentous fungi. PLoS ONE 10, e0133085. 26177455 10.1371/journal.pone.0133085 PMC4503723

[15] Kuivanen J , Mojzita D , Wang Y , Hilditch S , Penttilä M , Richard P and Wiebe MG (2012) Engineering filamentous fungi for conversion of d‐galacturonic acid to L‐galactonic acid. Appl Environ Microbiol 78, 8676–8683. 23042175 10.1128/AEM.02171-12 PMC3502896

[16] Pitt D and Mosley MJ (1985) Enzymes of gluconate metabolism and glycolysis in Penicillium notatum. Antonie Leeuwenhoek 51, 353–364. 4091540 10.1007/BF02275041

[17] De Ley J (1966) 5‐Ketogluconic acid reductase. Methods Enzymol 9, 200–203.

[18] Bausch C , Peekhaus N , Utz C , Blais T , Murray E , Lowary T and Conway T (1998) Sequence analysis of the gntii (subsidiary) system for gluconate metabolism reveals a novel pathway for l‐idonic acid catabolism in escherichia coli. J Bacteriol 180, 3704–3710. 9658018 10.1128/jb.180.14.3704-3710.1998 PMC107343

[19] Müller H‐M (1985) Utilization of gluconate by aspergillus niger. I. enzymes of phosphorylating and nonphosphorylating pathways. Zentralbl Mikrobiol 140, 475–484. 4072456

[20] Elzainy TA , Hassan MM and Allam AM (1973) New pathway for nonphosphorylated degradation of gluconate by aspergillus niger. J Bacteriol 114, 457–459. 4698214 10.1128/jb.114.1.457-459.1973 PMC251790

[21] Allam AM , Hassan MM and Elzainy TA (1975) Formation and Cleavage of 2‐keto‐3‐deoxygluconate by 2‐keto‐3‐deoxygluconate aldolase of Aspergillus niger. J Bacteriol 124, 1128–1131. 358 10.1128/jb.124.3.1128-1131.1975 PMC236016

