# Supplementary figures and images for: NADPH‐dependent 5‐keto‐D‐gluconate reductase is a part of the fungal pathway for D‐glucuronate catabolism
Source: FEBS Lett. 2017 Dec 30;592(1):71–7. doi: 10.1002/1873-3468.12946 (PMC5814732; doi:10.1002/1873-3468.12946)

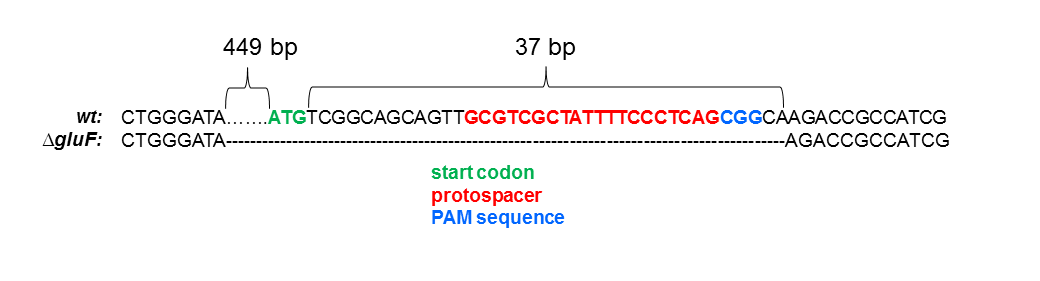

Supplement: Supplementary file 1 — Fig. S1. Disruption of the gluF gene after CRISPR/Cas9 genome editing. [file FEB2-592-71-s001.tif]

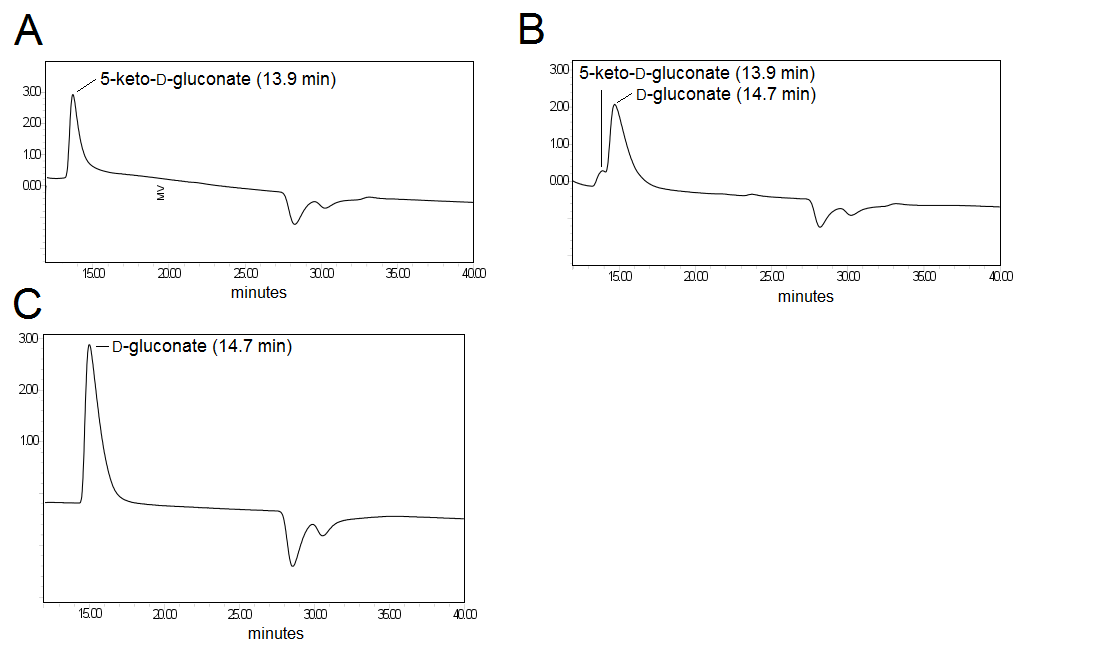

Supplement: Supplementary file 2 — Fig. S2. HPLC analysis of the reaction mixtures containing TRIS‐buffer, NADPH and 5‐keto‐D‐gluconate (A) without, (B) with the purified GluF protein and (C) D‐gluconate standard solution. [file FEB2-592-71-s002.tif]
